# 3D imaging using scanning diffractometry

**DOI:** 10.1038/s41598-020-79939-y

**Published:** 2021-01-12

**Authors:** Morteza J. Siavashani, Iman Naghedi, Vahid Abbasian, Ehsan A. Akhlaghi, Mohammad A. Charsooghi, Mohammad Taghi Tavassoly, Ali-Reza Moradi

**Affiliations:** 1 Department of Physics, Institute for Advanced Studies in Basic Sciences (IASBS), Zanjan, 45137-66731 Iran; 2 School of Nano Science, Institute for Research in Fundamental Sciences (IPM), Tehran, 19395-5531 Iran; 3 Optics Research Center, Institute for Advanced Studies in Basic Sciences (IASBS), Zanjan, 45137-66731 Iran; 4 Department of Physics, College of Science, University of Tehran, Kargar Shomali Ave, Tehran, 14399-55961 Iran

**Keywords:** Optical techniques, Imaging and sensing, Microscopy

## Abstract

Imaging of cells is a challenging problem as they do not appreciably change the intensity of the illuminating light. Interferometry-based methods to do this task suffer from high sensitivity to environmental vibrations. We introduce scanning diffractometry as a simple non-contact and vibration-immune methodology for quantitative phase imaging. Fresnel diffractometry by a phase step has led to several applications such as high-precision measurements of displacement. Additional scanning may lead to 3D imaging straightforwardly. We apply the technique to acquire 3D images of holographic grating, red blood cell, neuron, and sperm cell. Either visibility of the diffraction fringes or the positions of extrema may be used for phase change detection. The theoretical analysis through the Fresnel diffraction from one-dimensional phase step is presented and the experimental results are validated with digital holographic microscopy. The presented technique can be suggested to serve as a robust device for 3D phase imaging and biomedical measurements.

## Introduction

The imaging technologies based on different forms of energy interacting with tissues for non-invasive visualization of biomaterials have led by the interdisciplinary cooperation of physicists, biologists, engineers, physicians, etc^1^. Biomedical optical imaging is a rapidly emerging field with widespread applications ranging from clinical diagnosis to molecular biology^2^. Many of the ideas for cell imaging have been commercialized^3^; for example, fluorescent microscopes are commonly available in biology laboratories^4^. Fluorescent microscopy has been a versatile tool for live cell imaging, and further developments, such as the use of confocal microscopy^5^ and light sheet microscopy^6^ have been presented for high-throughput imaging. Optical imaging techniques possess several benefits; they can detect functional and structural changes with high sensitivity, they are non-invasive and real-time, and they can be set up portable with low equipment cost. The principal limitations of optical imaging techniques in biology and medicine are the limited resolution—due to Abbe’s diffraction limit^7^, limited penetration depth—due to the scattering of optical radiation in tissues^1^, and qualitative visualization in 3D - due to 2D nature of the methods^8^. A broad range of approaches has been proposed and developed during the last few decades to improve the imaging performance and several solutions have been presented to overcome the aforementioned limitations^9–15^.

The conventional microscopy techniques can detect only the intensity and the color of the light scattered from the sample, hence, 2D images are only acquired^4^. On the other hand, most of the biological specimens are relatively transparent, and for sufficient contrast, staining methods are used. For detecting the optical phase changes of the sample methods such as Zernike phase contrast microscopy^16^ and differential interference contrast microscopy^4^ are utilized. The problem with these methods is that they are inherently qualitative. However, quantitative phase imaging (QPI), through either raw data or statistical post-processing, provides high-content information about the phase and transparent samples^17^. The advancements of various QPI techniques have shown great potential for translation into the fields of cell biology, biophysics, and medicine^18^. QPI techniques show promises for the cellular-level study of cellular metabolism and activities in pathophysiology, which is crucial for understanding the mechanisms behind human diseases. The applications of QPI ranges from structures of cells and tissues, and cell dynamics, e.g. cell growth and division, to infectious diseases, e.g. genetic diseases and cancers^19,20^. Several important biological specimens have been investigated in vitro or ex vivo using QPI techniques, such as red blood cells, neuron cells in culture, and cardiomyocytes^21–23^. However, it is predicted that in the future, QPI techniques would be capable to study biological cells and tissues in vivo, as well^17^. QPI techniques employ mostly the principle of interferometry to measure the optical field, consisting of amplitude and phase information. There are two main classes of QPI techniques depending on their modulation: the spatial modulation or temporal modulation QPIs. In the spatial modulation scheme, a sample beam interferes with a reference beam and forms fringe patterns, from which the field information of the sample is retrieved. Temporal modulation, instead, records sequential interferograms by changing the phase of a reference beam with respect to a sample beam. For QPI several approaches have been reported, such as digital holographic microscopy^24,25^ (DHM), spatial light interference microscopy^26^, Spectral-domain phase microscopy^27^, and Fourier ptychographic microscopy^28,29^, to name a few. Non-interferometric QPI can also be performed which is based on the use transport of intensity equation^30^. Furthermore, QPI techniques can be integrated with the other optical methodologies, such as optical coherence tomography^31^, Raman spectroscopy^32^, fluorescence^21^, and polarization-sensitive measurements (for materials with birefringence such as spindle fibers and collagen fibers)^33^, to enhance further the capabilities of both integrating techniques.

Among the techniques to obtain a quantitative assessment of the optical phase changes, therefore, 3D imaging, DHM has been emphasized and developed more than others^24,25^. Several researches have been dedicated to DHM and its various applications, especially for non-invasive imaging of living cells and organelles^22,34^. Since DHM is considered as the combination of interferometry and optical microscopy, the functionality of the system highly depends on the fringes’ characteristics of the interference between the object and the reference waves. Environmental and mechanical noises may affect fringes dramatically. In order to reduce the noises, several common-path and self-referencing arrangements for DHM have been suggested and developed^35,36^. In such geometries, the noises associated with the object and reference waves are correlated. However, due to the overall instability of the optical system, the remained noises still affect the quality of the reconstructed images. Moreover, most of these arrangements work efficiently only if the sample under study contains large sparse domains^35^. For DHM experiments laser sources are commonly used, which results in speckle noises due to diffraction from every element along the optical train. In order to increase the quality of the holograms, utilization of low coherent sources or coherence reduction by rotating diffusers are suggested^37^. This, in turn, leads to the requirement of fine adjustment of the optical system to achieve high quality holography fringes.

In this paper, we provide a simple methodology for interference-free quantitative phase imaging through scanning diffractometry. When a light field passes through or is reflected from an object and experiences an abrupt change in its amplitude, phase, or polarization, it results in the formation of Fresnel diffraction patterns. The formed diffraction pattern includes information of the object which can be on its light absorption behavior, optical phase changes, or polarizing characteristics. The study of the diffraction pattern to extract such information of the object is known as “Optical Diffractometry (OD)”^38^. A sharp phase change (phase step) occurs when the light reflects from a physical step or passes through the boundary region of transparent media with different refractive indices. It has been shown that the visibility of the fringes of the diffraction from the phase step and the position of extrema vary by the change of the phase difference of the two sides of the phase steps^38–40^. This, in turn, leads to several interesting applications such as precise measurement of thin-film thickness^41^, refractive indices of solids and liquids^41–44^, nanometer displacements^45,46^, diffusion coefficient^38,47^, etching rate^48^, wavelength^47^, and coherence length and spectral line shape^49^. However, none of the aforementioned applications includes imaging of the objects. Here, we address the use of OD for 3D imaging of the samples and quantitative phase contrast microscopy.

## Theory and procedure

**Figure 1:**
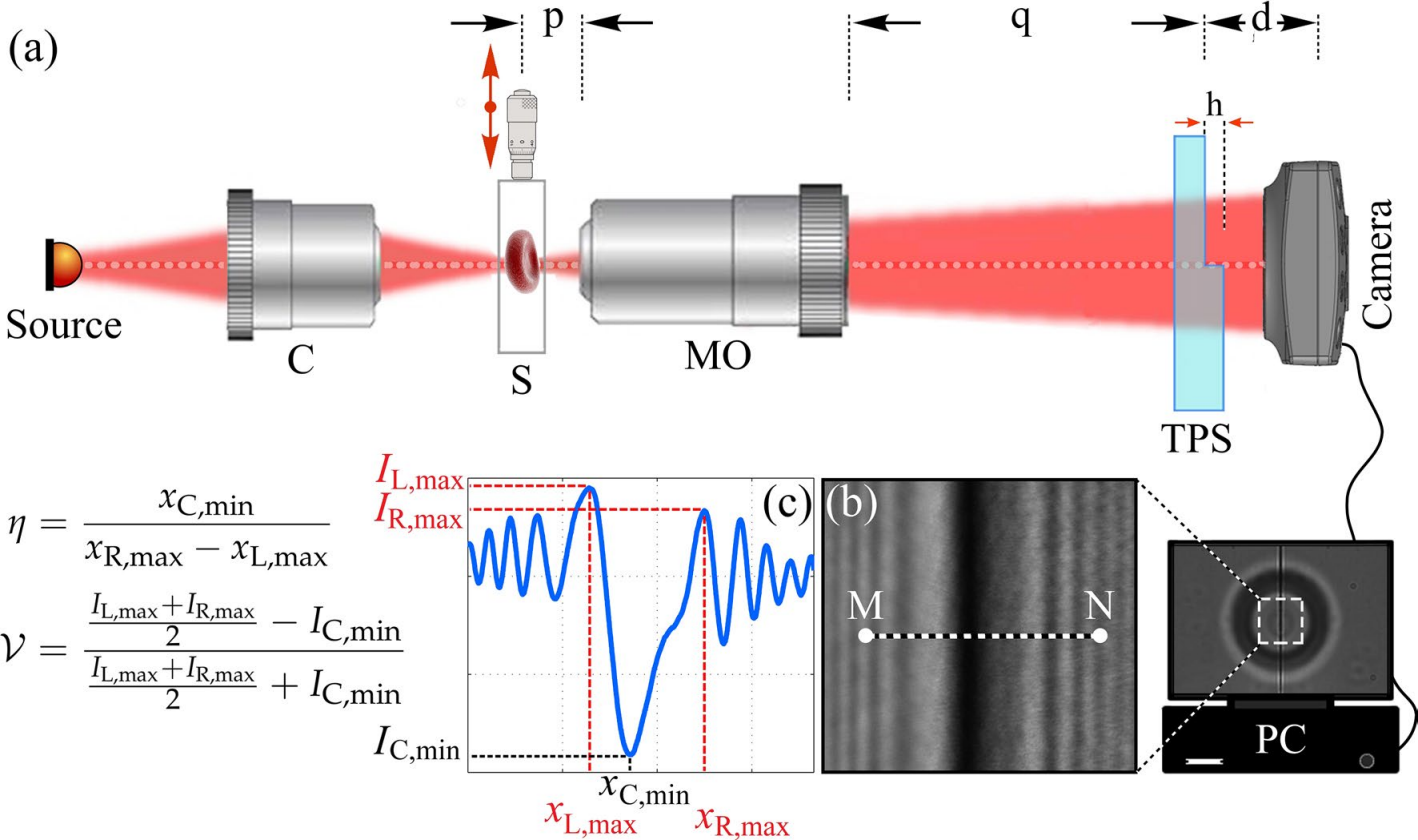
**(a)** The experimental setup for scanning OD; Source: low coherent LED, C: condenser, S: sample, MO: microscope objective, TPS: transparent phase step, h: height of the TPS, p: sample distance to MO, q: TPS distance to back aperture of MO, and d: TPS distance to camera. **(b)** Typical recorded diffraction pattern. **(c)** Cross-sectional profile along $\overline{\rm{MN}}$ in panel **(b)** and the definition of parameters $\mathcal {V}$ and $\eta$.

A simple setup for phase imaging based on the diffractometry method is shown in Fig. 1. The arrangement includes focusing a low-coherence beam through a condenser lens (C) onto the microscopic sample. The sample is mounted on a precise computer controlled translation stage (Luminos, I3005) that is used for the sample scanning procedure. The light diffracted from the part of the sample, which is crossing the optical axis, is collected by a microscope objective (MO, Olympus, UPLFLN, 40 $\times$, NA $=$ 0.75). The divergent beam from the MO carries the phase object information and reaches the camera (DCC1545M, Thorlabs, 8 bit dynamic range, 1024$\times$1280 pixels, 5.2 $\upmu$m pixel pitch) after passing through a transparent phase step (TPS) that is placed $d=5$ mm away from the camera. The TPS is parallel to the camera surface and its edge crosses the optical axis. The TPS is fabricated by coating a MgF$_2$ film on half of the surface of an optical plate through thermal physical vapor deposition. The thickness of the thin film is $h=2.2\pm 0.04$ $\upmu$m. The sample is placed around the focal plane of the MO ($p\simeq 550~\upmu$m). The distance between the back aperture of the MO and the TPS is about $q=30$ cm, which is the proper value to image the sample onto the TPS. Therefore, while the sample is scanned in front of the MO, the additional phase—imposed by the section of the object on the optical axis—converts the phase step into a phase step with a different height. The difference between the height of the virtual phase step and the TPS, therefore, corresponds to the phase change of the sample under study at the section crossing the optical axis. That phase will be extracted from the recorded Fresnel diffraction patterns. The distance between the TPS and its diffraction plane is chosen such that the diffraction pattern of the sample is located within the active area of the camera.

**Figure 2:**
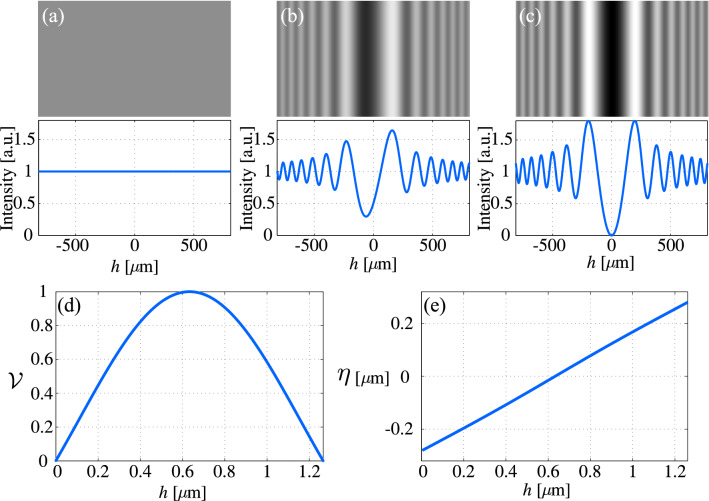
Simulations; Diffraction pattern of a phase step with the height of **(a)** 0, **(b)** 0.32 $\upmu$m, and **(c)** 0.63 $\upmu$m and their corresponding horizontal cross-sectional profiles. Wavelength is taken as 633 nm. **(d)** Visibility of the fringes, and **(e)** extrema position parameter vs. the phase step height variations in one period.

We extract the height difference of the virtual phase step and the TPS from the diffraction patterns through two different approaches; one of the approaches is based on the use of the intensity profile of the diffraction patterns, and the other one is based on the use of the positions of the extrema of the fringes. For a TPS of height *h* that is shone by a plane wave of intensity $I_0$, the intensity distribution of the Fresnel diffraction pattern at a plane perpendicular to the average propagation direction can be expressed as^50^:

1 $$\begin{aligned} I= & {} I_0 t_{\rm{L}} t_{\rm{R}} \left[ \left( \frac{1}{2}+C_0^2+S_0^2\right) +\left( \frac{1}{2}-C_0^2-S_0^2\right) \cos {\Delta \phi } - (C_0-S_0)\sin {\Delta \phi }\right] \nonumber \\&+ \frac{I_0}{2} \left[ \left( \frac{1}{2} + C_0^2+S_0^2\right) (t_{\rm{L}}-t_{\rm{R}})^2 + (C_0+S_0)(t_{\rm{L}}^2-t_{\rm{R}}^2)\right] , \end{aligned}$$

where $t_{\rm{L}}$ and $t_{\rm{R}}$ are the transmission coefficients of the left and right side of the step, respectively. $C_0$ and $S_0$ are the well-known Fresnel integrals^7^. Optical path difference between the lights from the left and right side of the TPS cause an abrupt phase change at the edge of the TPS:

2 $$\begin{aligned} \Delta \phi = \frac{2\pi }{\lambda } h n_{\rm{m}} \left[ \sqrt{\left( \frac{n_{\rm{TPS}}}{n_{\rm{m}}}\right) ^2-\sin ^2\theta _i} -\cos \theta _i \right] , \end{aligned}$$

where $n_{\rm{TPS}}$ and $n_{\rm{m}}$ are the refractive indices of the TPS material and the surrounding medium, respectively, $\lambda$ is the wavelength of the incident light, and $\theta _i$ is its angle of incidence. Changes in any of the parameters related to the physical properties of TPS will lead to a substantial change in the intensity profile of the diffraction pattern. For simplicity, we consider a TPS with identical left and right transmission coefficients surrounded by air. Figure 2a–c show the simulated diffraction patterns and their corresponding averaged cross-sectional profiles of phase steps with the heights of 0, 0.32 $\upmu$m, and 0.63 $\upmu$m, respectively. We define the visibility of the diffraction fringes as following^38^:

3 $$\begin{aligned} {\mathcal {V}} = \frac{\frac{I_{{\hbox {L,max}}}+I_{{\hbox {R,max}}}}{2}-I_{{\hbox {C,min}}}}{\frac{I_{\hbox {{L,max}}}+I_{\hbox{{R,max}}}}{2}+I_{{\hbox {C,min}}}}, \end{aligned}$$

where L, R, and C stand for left, right, and center of the fringes, respectively, and $I_{\rm{max}}$ and $I_{\rm{min}}$ are the absolute maximum and minimum intensities of the patterns, respectively. Figure 2d shows the variation of the visibility as a function of the TPS height. The visibility versus height is a periodic function and only one period is shown here. The maximum visibility belongs to the states that all the involving parameters e.g. physical height, refractive index, and the incident angle, cause phase differences of $\Delta \phi =(2M+1) \pi$, *M* being an integer. The minimum visibility, on the other hand, corresponds to $\Delta \phi =(2M) \pi$. The curve is used to measure the phase changes, and if the other parameters are known, the obtained phase can be treated similarly to the fringe analysis in interferometry for the height change determination. If the height change brings $\mathcal {V}$ into the range of [0, $\mathcal {V}{\rm{max}}$] in the first period, a unique phase and height change will be extracted. If $\mathcal {V}$ falls in the second half-period, two different phases are obtained. However, according to the asymmetric nature of the phase step diffraction pattern the actual phase can be uniquely determined. Higher changes of height, in the other $\mathcal {V}$ periods, can be extracted by proper unwrapping process^51^.

An additional benefit of OD based on the use of TPS is that even for a TPS with specified physical parameters, by proper choosing of the incident angle of light the desired phase changes can be applied. Therefore, for example, the configuration may be arranged so that the linear part of the visibility curve is taken to use. In our setup, this is simply done by rotating the TPS around the axis perpendicular to the optical axis and parallel to the edge of the TPS.

The visibility parameter to determine the height change is based on the variations of the intensity profile of the diffraction pattern. The precision of the approach, therefore, depends on the sensitivity of the detector. Due to the availability of highly sensitive cameras in the market, the precision can be very high. However, on the other hand, the dependence on the intensity variations requires high stability against spatial and temporal variations of the intensity arriving at the detector. Here, we define other parameters for height measurement through the analysis of the phase step diffraction pattern that are not based on the intensity variations; these parameters, instead, are based on the position of the extrema of the patterns. The diffraction pattern of a TPS has an absolute minimum and two absolute maxima in the right and left side of the absolute minimum. Also, there are several consequent minima and maxima in the two sides. Therefore, the positions of these extrema points provide plenty of parameters that can be used to determine the associated phase changes of the TPS. We have examined several parameters, and amongst, the following parameter, which we name as “Extrema position parameter”, is proved as a suitable parameter for height change determination:

4 $$\begin{aligned} \eta = \frac{x_{\rm{C,min}}}{x_{\rm{R,max}} - x_{\rm{L,max}}}, \end{aligned}$$

where, $x_{\rm{min}}$ and $x_{\rm{max}}$ are the positions of the fringe minimum and maxima. Figure 2e shows the variations of $\eta$ versus height change in one period, which, up to a high extent, is linear. In order to calibrate $\eta$ for a specific diffractometry setup, the slope and the intercept have to be determined. The slope can be determined by $x_{\rm{R,max}} - x_{\rm{L,max}}$, which is shown to be almost a constant against height variations. Therefore, the phase change determination procedure will not be dependent on this parameter. The intercept of the calibration graph may be determined by proper positioning of the center of the camera.

## Results and discussion

**Figure 3:**
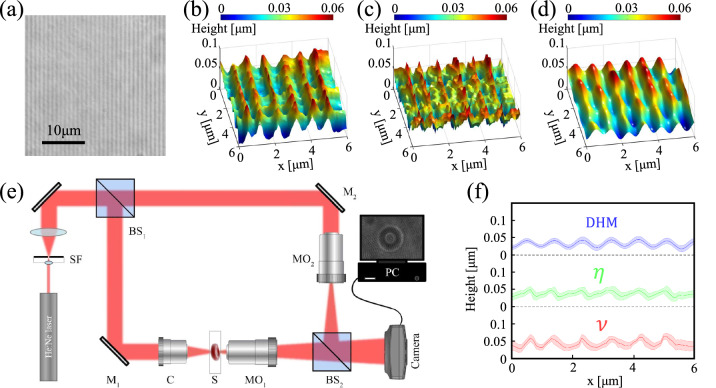
Experimental results for a transparent grating; **(a)** Micrograph of the grating. **(b)** The reconstructed 3D image of a grating obtained by the phase step through visibility parameter, and **(c)** through extrema position parameter. **(d)** DHM reconstructed image. **(e)** A schematic of the Mach–Zehnder DHM setup, used for verification experiments; **(f)** averaged cross-sectional profile of the grating for the three approaches. Shaded errorbars indicate the standard deviation of the data.

**Figure 4:**
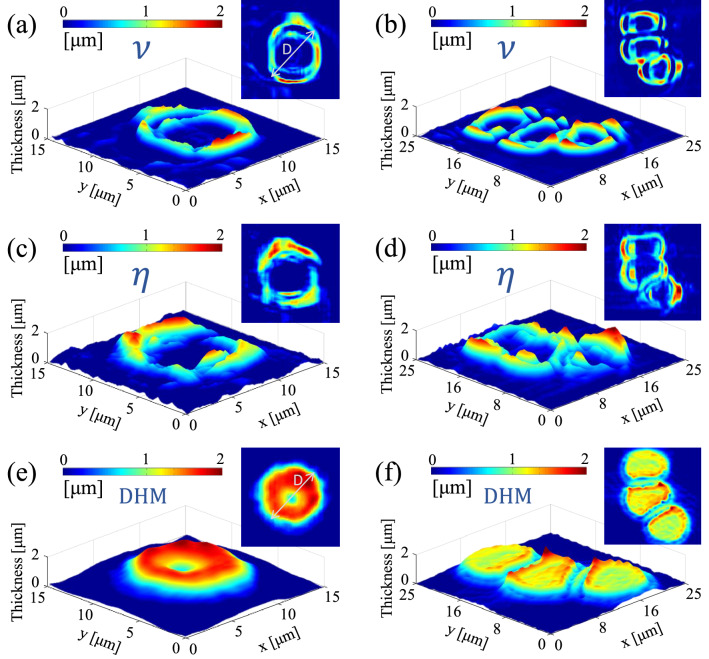
Experimental results for RBCs; the reconstructed 3D images and 2D maps of (left) a single RBC and (right) multiple RBCs, obtained by **(a,b)** phase step through $\mathcal {V}$, **(c,d)** phase step through $\eta$, and **(e,f)** DHM method. In panels **(a,c)** *D* shows the lateral diameter of the RBC and its average is reported for quantitative assessment of the OD method.

**Figure 5:**
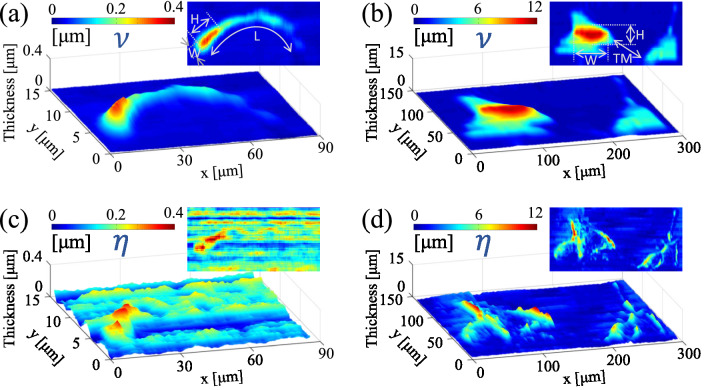
Experimental results for neurons (U87) and bovine sperm cells; Reconstructed 3D images of a dense sample of neurons obtained through **(a)** $\mathcal {V}$ and **(b)** $\eta$ parameters. Reconstructed 3D images of a sperm cell through **(c)** $\mathcal {V}$ and **(d)** $\eta$ parameters. The insets are the cross-sectional profiles along $\overline{\rm{AB}}$ and $\overline{\rm{A}'\rm{B}'}$ lines depicted in panels **(c,d)**, respectively. In panels **(a,b)** the cells typical dimensions are shown.

The experimental examination of the present phase imaging approach for various categories of phase objects including a holographic grating, RBC, neuron, and sperm cells are presented in the following. In Fig. 3 resulted height profile of a 1000 lines/mm transparent grating is shown. A micrograph of the grating is shown in Fig. 3a–c represent the reconstructed topographic images obtained by $\mathcal {V}$ and by $\eta$, respectively. In order to verify the results, we have conducted controlling experiments with a Mach–Zehnder based DHM setup that is a commonly used quantitative phase imaging technique. The corresponding DHM result for the grating is depicted in Fig. 3d, and the schematic arrangement of the DHM setup is shown in Fig. 3e. The holograms are reconstructed by the angular spectrum propagation method^52^. Figure 3f shows the averaged cross-sectional profiles of the grating along x-axis direction. The standard deviation of the acquired data is also shown as the shaded errorbar. The results confirm the capability of the diffractometry approach for 3D imaging of a periodic phase object such as the transparent grating; the average distance between two adjusted lines in both methods are obtained about $1\,\upmu \rm{m}$. We further apply the technique for arbitrary shape phase objects. Figure 4 shows the reconstructed 3D images of RBC samples. In order to confirm that the method is capable of using for crowded samples as well, we also perform experiments on the RBC samples that include several cells in the field of view. The reconstructed 3D images and 2D maps of single RBC and multiple RBCs obtained through $\mathcal {V}$ parameter are depicted in Fig. 4a,b, respectively. The corresponding results obtained through $\eta$ parameter and from DHM experiments are shown in panels c,d and e,f, respectively. The results of the OD approach ($\mathcal {V}$ parameter) are in agreement with the results of DHM for the size and the hight of the cells. However, 3D images reconstructed by DHM represent better quality than OD, especially in the middle of the cells. The average lateral diameter of the single RBC (*D* in Fig. 4a,e) obtained by the OD method is $8.9\,\upmu \rm{m}$, which is comparable with the DHM result which is $8.7\,\upmu \rm{ m}$. The OD and DHM methods provide the maximum thickness of the cell as $1.9\,\upmu \rm{m}$ and $1.8\,\upmu \rm{m}$, respectively, which are in a satisfactory agreement. The fact that OD approaches provide acceptable results for a dense sample is of high significance; because, according to the nature of the method, every part of a sample takes part in the overall diffraction pattern. The effective height of the TPS consists of its physical height and the thickness of the sectional part of the sample that is crossing the TPS during the scanning process. The influence of the rest of the sample away from the TPS edge on the diffraction pattern is negligible. Figure 5 shows the reconstructed 3D images of sperm and neuron cells. Similar to several biological specimens these cells are also relatively transparent, and their quantitative phase imaging may provide valuable morphometric information on their behavior, status, and reaction to external stimuli and factors. For example, the response of a neuron to an external electric or chemical stimulus can be appeared as a change on its morphology. The detection of this change by quantitative phase imaging approaches, such as DHM and OD provides a reliable assessment procedure on cell analysis in drug effect studies and neurobiology investigations. In^53^, for example, quantitative phase imaging through DHM is used to resolve neuronal network activity and identify cellular biomarkers of psychiatric disorders. In panels a and b the cells typical dimensions are shown. Despite the images obtained from $\mathcal {V}$ parameter (Fig. 5a,b, respectively), which show satisfactory results, there is a failure for the reconstructed 3D images from $\eta$ parameter (Fig. 5c,d). This is attributed to the different nature of the two approaches. In the former one, the bit depth of the imaging camera plays the main role in the final resolution, whereas in the latter one the pixel size of the camera is important. For neuron and sperm samples, especially for the narrower parts of them, the influence on the extrema positions from the sample cannot be recognized in the diffraction pattern due to the relatively large pixel size of the sensor comparing to its high dynamical range. The total length of bovine sperm cell (Fig.5a) is obtained $\rm{L}=89.5\,\upmu \rm{m}$, and its head dimensions are $\rm{W}=22.5\,\upmu \rm{m}$ and $\rm{H}=9.8\,\upmu \rm{ m}$. For the U87 cell (Fig. 5b), we obtained body sizes as $\rm{W}=84.0\,\upmu \rm{m}$, $\rm{H}=88.5\,\upmu \rm{m}$, and axon length as $\rm{TM} = 67.05\,\upmu \rm{m}$, which are in agreement with the sizes provided by the American Type Culture Collection (ATCC)^54^.

The accuracy of the presented method can be affected by the resolution of the scanning device (100 nm), which in turn affects the diffraction pattern, response of the recoding sensor to light, and the aberrations and contaminations that might be present in the elements of the optical train, which, indeed, is present in all microscopic imaging modalities and its effect is minimized by the use of the aberration-corrected microscope objectives. According to the results of the standard sample examination (Fig. 3), in lateral direction, the reconstructed results for both parameters are in a good agreement with the characteristics of the grating. For the out-of-plane direction, DHM as a standard quantitative and morphometric phase imaging system, is taken as verifying alternative method. In some cases, such as RBCs the results have moderate agreement with DHM results, which we attribute it to the phase wrapping problem within the reconstruction process. However, the $\mathcal {V}$-based results of the grating, sperms and neuron cells are in agreement with the DHM results and the typical data provided by the common sources for this type of cells (American Type Culture Collection), respectively. The $\eta$-based results fail in the cases of slowly varying thickness, such as the tail of the sperm cell and neuron cell body. Considering the $\mathcal {V}$-based results for the grating, we estimate the axial and lateral accuracies of the current OD system as 30 nm and 100 nm, respectively. The laser intensity fluctuations as well as the possible non-uniformity of the applied phase step may cause random errors and affect the reliability of the results. We deal with this issue by averaging over a neighboring pixels along the lines perpendicular to the phase step edge. This may cause loosing some data, but, instead, the results will be more reliable and smoother. According to the simple nature of the experimental apparatus, to be assured of the reliability, the experiments can be easily repeated for the samples and the average results can be considered as the reported one.

According to the results on several samples, which are inherently different from each other in their shape and structure, OD offers a capable imaging technique to obtain 3D quantitative images of a large class of phase objects including biomaterials and cells. The morphological information, in turn, may be subjected to proper data analysis procedures. For example, detection of local fluctuations of cell membrane which are easily measured by the OD method can be used for acquiring information on the rigidity and morphology features of the cells. However, it should be noted that according to the scanning nature of the method the samples have to be immobilized or the dynamicity of them should be slower than the scanning speed. Due to the structure of the scanning phase step and the possibility of averaging along the direction of the edge of phase step it is clear that the methodology may be more reliable for the samples with a symmetry in one direction. A remarkable feature of the method, despite to probe-based imaging approaches is that the OD approach can be also applied in the cases that frequent singularities and defects are present in the sample. The method can be applied for the objects of various lateral size within an adjustable scanning resolution. The non-interferometric nature of the method ensures of its immunity against environmental noises. Moreover, despite the interferometric techniques with partially coherent sources that come along with limited fringe covering area, in OD the fringes around the part of the object crossing the phase step edge is sufficient for the measurements.

Since the precision of the common scanning devices is much beyond the diffraction limit, the lateral resolution of the acquired images of this method is dictated by the resolution of the optical system. Therefore, the lateral resolution enhancement methods can also be integrated with this 3D imaging technique. In this research, the lateral resolution of the OD method, i.e., the translation stage step is $0.1\,\upmu \rm{m}$. The consequence of the scanning procedure is that a wide field of view can be imaged, inside the range of the scanning device and the initial field of view of the optical system. However, in the case of 3D image reconstruction through $\eta$ parameter, since it is based on the relative position of extrema, the pixel size of the recording digital camera may affect the precision of the final image. It is shown, both theoretically and experimentally, that in OD methodology, according to the nature of the method, variations in optical path difference down to $0.01\,\upmu \rm{m}$ can be feasibly detected using visible light sources^41,43,45^. Furthermore, the axial resolution in shorter wavelengths, e.g., in X-ray regime^44^, can be even better.

The presented method may be extended to reflective mode, simply by the use of a beam splitter to illuminate the sample, and behaving the returned light from the sample as the transmitted light of Fig. 1. However, according to the low working distance of microscope objectives, an imaging lens before the objective will be required.

Our technique possesses the main capabilities of other QPI techniques. It is non-invasive and label-free and has no need to use dye or fluorescent proteins for biological samples, exhibits no phototoxicity and photobleaching, and can be easily extended with other optical imaging modalities or time resolved techniques. The sensitivity of the method, due to the large slope magnitude of the visibility vs. phase change curve and similar to most of the other QPI techniques, is in nano scales and within the scanning speed limit can be used for dynamic phenomena. Furthermore, despite most of the interferometry-based QPI methods, the implementation of QPI techniques toward the easy usage by non-specialists is straightforward as the apparatus includes a single arm and a few optical elements. According to the simplicity of the setup, it has the possibility to be even miniaturized which potentially enables being set up in a portable platform. Nevertheless, the OD method is suggested as an alternative to other phase imaging methods such as DHM, and the aforementioned benefits, drawbacks and limitations are present similar to any other method.

An important feature of OD is that the methodology is somewhat independent of the source wavelength. It has been shown that partially coherent and very low coherent sources can also provide high contrast phase step fringes^41^. Further, the method can be also used for X-ray and acoustic sources, which in turn might find numerous applications in many areas such as biomedical imaging and geological studies.

The present method is a non-interferometric QPI technique, and for the first time, to the best of our knowledge, uses diffraction pattern for 3D imaging. Therefore, it does not suffer from time-varying phase noise due to vibration, temperature gradient, and air flow, which deteriorate the stability of QPI measurements.

## Conclusion

In conclusion, we introduce an interference-free and highly sensitive quantitative phase imaging (QPI) method based on Fresnel diffraction from a phase step. The preliminary results of the application of the method for several micro-objects including holographic grating and biological samples such as RBC, neuron, and sperm cell are presented. The optical diffractometry patterns provide several parameters to assess quantitatively the topological information of the samples. Amongst, visibility and extrema relative positions within the diffraction pattern are the proper choices to reconstruct the 3D images. The results are in agreement with the theoretical predictions and the controlling experimental results obtained by digital holographic microscopy as a well-established 3D imaging method. QPI techniques show promises for the cellular-level study of cellular metabolism and activities in pathophysiology. Most of the QPI techniques employ the principle of interferometry to measure the optical field, while the presented technique is based on diffraction. Yet, it possesses the main capabilities of other QPI techniques. Our method provides a wide field of view, and high and tunable lateral resolution for 3D imaging and is capable of integration with other optical techniques such as optical and acoustical tweezers, fluorescence microscopy, and resolution enhancement approaches. However, dynamicity and mobilization of the sample under study should be slower than scanning speed. According to the simplicity of the setup, it has the possibility to be miniaturized which potentially enables being set up in a portable platform. Therefore, the methodology can be suggested to serve as a bench-top vibration-immune device for 3D imaging and can be applied in various applications including biomedical measurements. It is remarkable that due to the diffraction-based nature of the OD method, it can be used for a variety of light sources from X-ray to infrared regions and also for acoustic sources. These extensions and the direct applications of the method to some biological phenomena are the subjects of some of our in progress follow-up researches.

## Methods

### Sample preparation

Red blood cells (RBCs) are obtained from the Blood Bank of Zanjan, Iran, and from freshly collected human blood, drawn from clinically healthy donors, from whom informed consent was obtained in accordance with the Blood Bank regulations. The plasma and buffy coat of fresh blood are separated by centrifugation at 3000 g for 10 min at 4 $^{\circ }$C temperature, and the separated layers are removed by careful aspiration. The cells are resuspended in physiological solution (NaCL,150 mM), and are washed three more times with the same buffer, in order to obtain a 0.1% hematocrit value, which is the suitable concentration for microscopic experiments that require a single-cell or few cells in the field of view. The RBC specimens are kept in water bath at 37 $^{\circ }$C before the experiments. In the experiments, a microliter of the diluted blood is smeared on a single glass microscope slide to produce a thin film. Slides are air-dried for 20 min. The experimental protocols are conducted in accordance with the regulations and policies and under approval of the Blood Bank of Zanjan, which are also in accordance with the regulations under WMA Declaration of Helsinki.

Cryopreserved cells (cell line code: U87) are unfrozen and cultured in a flask for propagation in a medium recommended by American Type Culture Collection (ATCC). The culture medium consists of 10% FBS and the rest is filled with DMEM. Cells are cultivated in 25 ml filtered flasks and passaged every 2 days (PBS as washing agent and trypsin as a de-attaching agent are used in the order). Incubation conditions are as follows; 5% CO$_2$ atmosphere and 37$^{\circ }$C temperature. Third generation passaged cells are used. As flasks are transparent, we use the flask itself for imaging purposes.

The frozen bovine sperm cells in liquid nitrogen are unfrozen by a water bath at $38^{\circ }C$. The sperm cells are diluted 1:100 (v:v) in 200 mM Tris, 65 mM citric acid, and 55 mM glucose solution. A drop of diluted sperms is injected inside a glass slide, which is covered with a coverslip (20 mm $\times$ 20 mm) and linked to the glass slide by double-stick tapes. Two free sides of the chamber are sealed with petroleum jelly to avoid evaporation of the medium.
